# Mechanical and Hemodynamic Effects of Balloon-Expandable and Self-Expanding Stents in a Saccular Abdominal Aortic Aneurysm Model: Fluid–Structure Interaction Analyses

**DOI:** 10.3400/avd.oa.26-00052

**Published:** 2026-10-23

**Authors:** Masanori Murakami, Fei Jiang, Tatsuo Yamashita, Shinsuke Mochizuki

**Affiliations:** 1 Department of Cardiovascular Surgery, National Hospital Organization Kanmon Medical Center, Shimonoseki, Yamaguchi, Japan; 2 Department of Mechanical Engineering, Graduate School of Sciences and Technology for Innovation, Yamaguchi University, Ube, Yamaguchi, Japan

**Keywords:** abdominal aortic aneurysm, balloon-expandable stent, self-expanding stent, fluid–structure interaction, hemodynamics

## Abstract

**Objectives:**

The aim of this study was to compare the effects of balloon-expandable and self-expanding stents on vessel wall mechanics and hemodynamics in a patient-specific saccular abdominal aortic aneurysm model.

**Methods:**

A patient-specific aneurysm model was constructed using computed tomography images, and balloon-expandable and self-expanding stent models were created. Aortic wall stress was evaluated by contact analysis. Fluid–structure interaction analyses were performed at 5 representative phases selected from the cardiac cycle. Mean intra-aneurysmal flow velocity, wall shear stress (WSS), pressure loss, and energy loss were compared.

**Results:**

The balloon-expandable stent produced greater localized aortic wall stress than the self-expanding stent, whereas wall stress was more uniformly distributed with the self-expanding stent. Both stents reduced the mean intra-aneurysmal flow velocity, WSS, pressure loss, and energy loss compared with the no-stent model. However, greater reductions in WSS, pressure loss, and energy loss were noted with the balloon-expandable stent than with the self-expanding stent.

**Conclusions:**

Balloon-expandable and self-expanding stents exhibited different effects on vessel wall mechanics and hemodynamics. The findings indicate the importance of evaluating both hemodynamic and mechanical effects when assessing stent–vessel interactions.

## Introduction

Stents interact mechanically with the vessel wall according to their structural and mechanical properties, thereby altering vessel geometry and the mechanical loading of the vessel wall after deployment.^1,2)^ Changes in vessel geometry may also affect local hemodynamics.^3)^ Because balloon-expandable and self-expanding stents differ in their structural and mechanical properties, they may have different effects on both the vessel wall and local blood flow.^1,2)^

We previously used patient-specific models of saccular abdominal aortic aneurysms derived from computed tomography images to investigate, in a stepwise manner, hemodynamic changes associated with aneurysm geometry, the effects of stent placement on the vessel wall and blood flow, and the effects of different stent diameters and lengths.^4–6)^ Our initial study showed changes in flow velocity, wall shear stress (WSS), pressure, energy loss, and the pressure-loss coefficient during simulated growth of a saccular aneurysm.^4)^ We subsequently used fluid–structure interaction (FSI) analysis of stented models to evaluate changes in the vessel wall and blood flow after stent placement, including renal artery flow when the stent crossed the renal artery.^5)^ We further compared models with different stent diameters and lengths to examine the relationship between stent size, vessel wall mechanics, and hemodynamics.^6)^

In the present study, balloon-expandable and self-expanding stents were placed in the same saccular abdominal aortic aneurysm model and compared using identical vessel geometry and analysis conditions. Using contact analysis and FSI analysis, we aimed to determine how the 2 stent types differed in their effects on post-deployment vessel geometry, mechanical loading of the vessel wall, and hemodynamic characteristics.

## Materials and Methods

We constructed a saccular abdominal aortic aneurysm model based on computed tomography (CT) images and created models with balloon-expandable and self-expanding stents. The original aortic model without a stent served as the no-stent reference model. The mechanical effects of stent placement on the aortic wall were evaluated using contact analysis, and FSI analyses^7)^ were performed at 5 representative phases selected from the cardiac cycle. The overall workflow of the analysis is shown in **Supplementary Fig. 1**.

### Creation of an aortic model

A three-dimensional aortic model was constructed based on CT images from a case involving a saccular abdominal aortic aneurysm located below the renal artery. The image data (Digital Imaging and Communications in Medicine; Medical Imaging and Technology Alliance, Arlington, VA, USA) were imported into the three-dimensional image processing software Simpleware ScanIP version 2019 (Synopsys, Sunnyvale, CA, USA) to build the model. Overall, the aortic model was 67 mm long, with an aortic diameter of 17–18 mm around the aneurysm, a maximum aneurysm diameter of 28 mm, and a uniform wall thickness of 2 mm (**Fig. 1A**).

**Figure 1:**
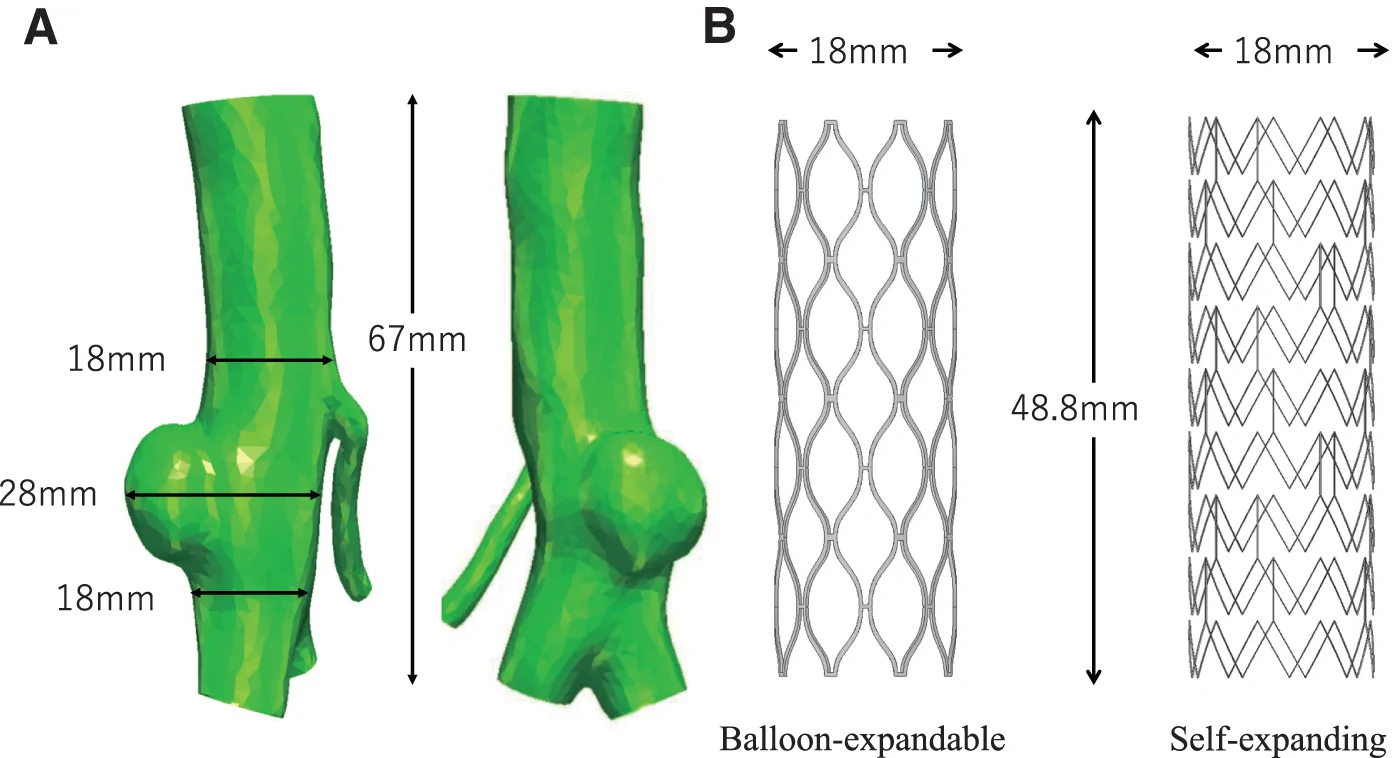
Fig. 1 Models used in the study. (**A**) Aortic model: total length, 67 mm; aortic diameter near the aneurysm, 17–18 mm; diameter of the aneurysm, 28 mm; wall thickness, 2 mm. (**B**) Stent models: left, balloon-expandable stent; right, self-expanding stent. Both stents were 48.8 mm long and 18 mm in diameter. The wire thicknesses were 0.3 and 0.1 mm for the balloon-expandable and self-expanding stents, respectively.

### Design and construction of stent models

The geometry of the balloon-expandable stent model was based on the Palmaz stent,^8)^ whereas that of the self-expanding stent model was based on the Epic Self-Expanding Nitinol Stent System.^9)^ Both stent models were created using the three-dimensional computer-aided design software Autodesk Inventor Professional 2020 (Autodesk, San Francisco, CA, USA). The stents were designed with a length of 48.8 mm, extending 1 cm beyond the aneurysm both proximally and distally, and a diameter of 18 mm to match the aortic diameter. The wire thicknesses were 0.3 and 0.1 mm for the balloon-expandable and self-expanding stents, respectively (**Fig. 1B**).

### Contact analysis, stent placement models, and creation of a blood model

The models were imported into Simpleware ScanIP and meshed with tetrahedral elements. Subsequently, the aortic and stent models were aligned to determine the stent’s placement within the aorta. After alignment, the meshed models were imported into Marc and Mentat version 2019 (Hexagon, Stockholm, Sweden). To simulate stent deployment, external compressive forces were applied to the stent and then released, enabling it to expand against the aortic wall. A contact analysis was subsequently performed. The material properties used in the contact analysis were as follows: the aortic wall was modeled as aortic tissue with a Young’s modulus of 0.5 MPa, a Poisson’s ratio of 0.49, and a density of 1000 kg/m³. Both stents were modeled as nitinol; the balloon-expandable stent had a Young’s modulus of 21000 MPa, whereas the self-expanding stent had a Young’s modulus of 10000 MPa. The Poisson’s ratio and density of both stents were 0.33 and 6450 kg/m³, respectively^10,11)^ (see **Supplementary Table 1**). Based on the deformed configuration obtained after contact analysis, composite models of the aorta and the stent—referred to as the post-stent placement models—were constructed (**Fig. 2A**). The mesh data from the contact analysis were reimported into Simpleware ScanIP, and the vascular lumen (inner cavity) was extracted from both the aortic and post-stent placement models. A fluid domain (blood model) was then created by filling this lumen with blood (**Fig. 2B**).

**Figure 2:**
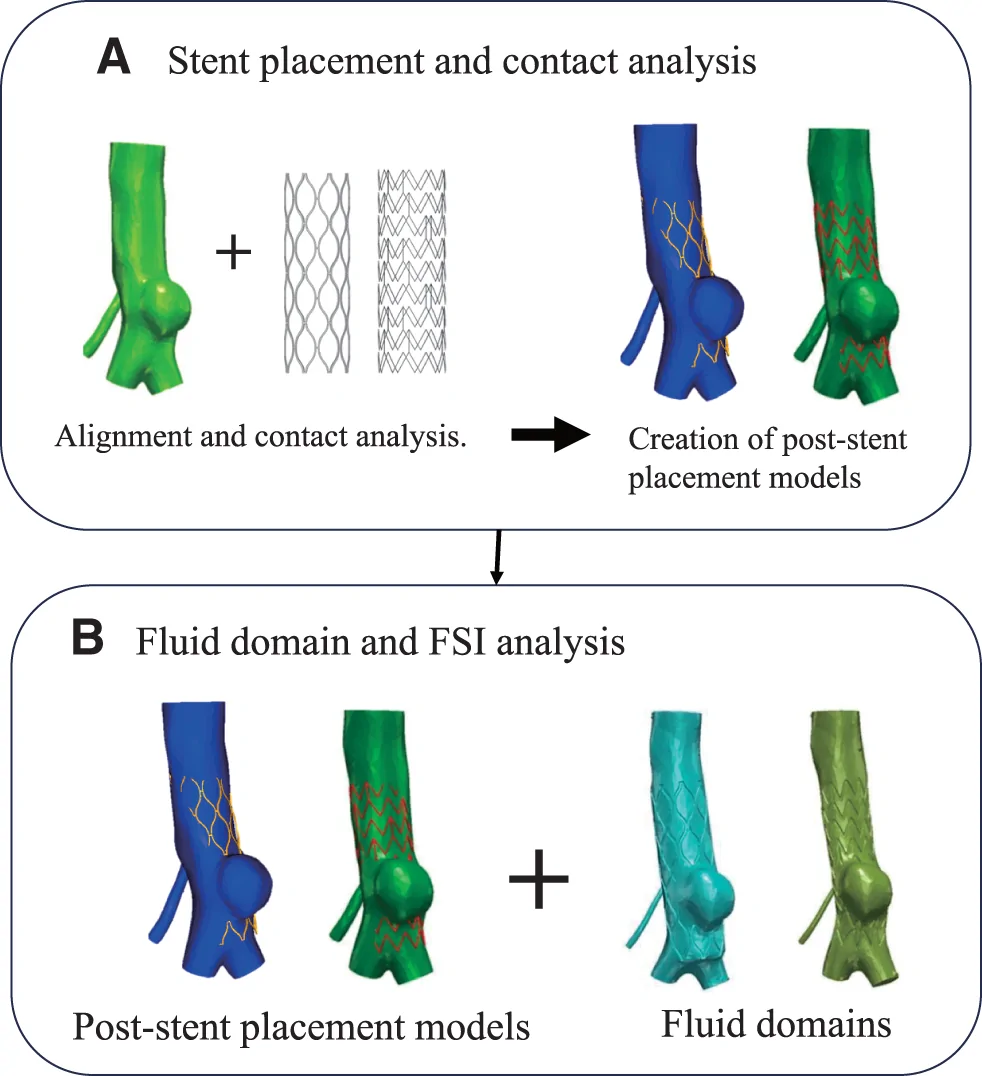
Fig. 2 Analysis process. (**A**) The aortic aneurysm models were created from computed tomography images, and the stent designs were created using three-dimensional computer-aided design software. The aortic and stent models were aligned, and stent deployment was simulated. Contact analysis was performed to evaluate the mechanical effects on the aortic wall, after which the post-stent placement models were constructed. (**B**) The lumen of the post-stent placement model was filled to create a blood model. FSI analysis was then conducted using the post-stent placement and blood models. FSI, fluid–structure interaction

### FSI analysis

Bidirectional FSI analysis was performed using COMSOL Multiphysics (Comsol, Burlington, MA, USA) to evaluate aortic wall deformation caused by blood pressure and its resulting effects on blood flow. In this study, the aorta and stent were modeled as a unified structure with the following material properties: a Young’s modulus of 0.5 MPa, a Poisson’s ratio of 0.49, and a density of 1000 kg/m³. Blood was modeled with a viscosity of 0.0045 Pa·s and a density of 1050 kg/m³. The inlet boundary was defined at the proximal abdominal aorta, and the outlet boundaries were defined at the inferior mesenteric artery and the bilateral common iliac arteries (**Fig. 3A**). The inlet and outlet pressure waveforms during 1 cardiac cycle are shown in **Fig. 3B**. To incorporate changes in the pressure difference during the cardiac cycle, 5 representative phases were selected based on a previously reported pressure-difference waveform: the initial increase, mid-ascending phase, peak difference, mid-descending phase, and terminal decline (**Fig. 3C**).^12)^ The pressure difference at each phase was applied as a boundary condition, and an individual FSI analysis was performed for each phase.

**Figure 3:**
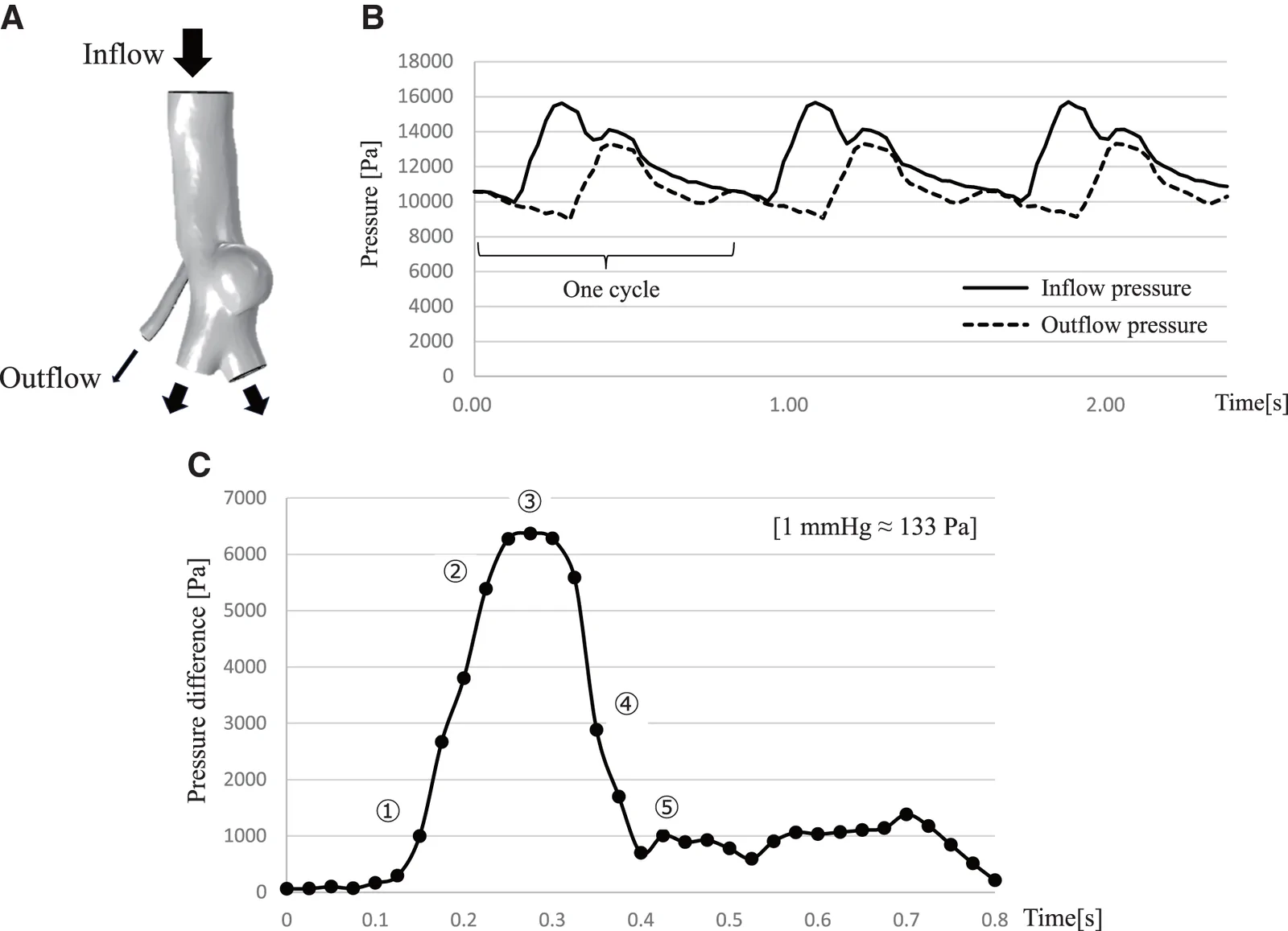
Fig. 3 Analysis conditions. (**A**) The inlet was defined at the proximal abdominal aorta, and the outlets were defined at the inferior mesenteric artery and the bilateral common iliac arteries. (**B**) Inlet and outlet pressure waveforms during 1 cardiac cycle. (**C**) Pressure difference between the inlet and outlet during 1 cardiac cycle. Five representative phases were selected: (1) initial increase, (2) mid-ascending phase, (3) peak difference, (4) mid-descending phase, and (5) terminal decline.

### Mechanical and hemodynamic evaluation indices

The following mechanical and hemodynamic indices were calculated and compared between the balloon-expandable and self-expanding stent models. Von Mises stress and circumferential stress were obtained from the contact analysis performed using Marc and Mentat. Streamlines, mean intra-aneurysmal flow velocity, WSS, pressure loss, and energy loss were obtained from the FSI analyses and post-processing using COMSOL Multiphysics. Mean flow velocity was evaluated within the aneurysm sac, whereas mean WSS was evaluated over the aneurysmal wall. Pressure loss^13,14)^ and energy loss^15)^ were calculated based on computed pressure and velocity distributions.

Von Mises stress: A scalar measure representing the magnitude of stress generated in the aortic wall during stent deployment.

Circumferential stress: Tensile or compressive stress acting along the circumferential direction of the aorta.

Streamlines: Visualization of the direction of blood flow within the model.

Mean intra-aneurysmal flow velocity: The mean blood flow velocity within the aneurysm sac.

WSS (mean WSS): The tangential frictional force exerted by blood flow on the vessel wall. In this study, mean WSS was evaluated over the aneurysmal wall.

Pressure loss: The decrease in pressure as blood flows through the analyzed vascular segment.

Energy loss: The energy lost as blood flows through the analyzed vascular region because of factors such as blood viscosity and flow disturbance.

The study protocol was reviewed and approved by the Institutional Ethics Committee of the National Hospital Organization Kanmon Medical Center (Approval No. R0202-2) on March 30, 2020. The requirement for informed consent was waived because only anonymized clinical imaging data were used.

## Results

### Contact analysis

In the balloon-expandable stent model, von Mises stress increased in the aortic wall at the stent implantation site, with localized stress concentrations particularly at regions of vessel curvature and diameter transition. In contrast, the self-expanding stent conformed more closely to the vessel geometry, and the increase in von Mises stress was smaller than that observed with the balloon-expandable stent (**Fig. 4A**).

**Figure 4:**
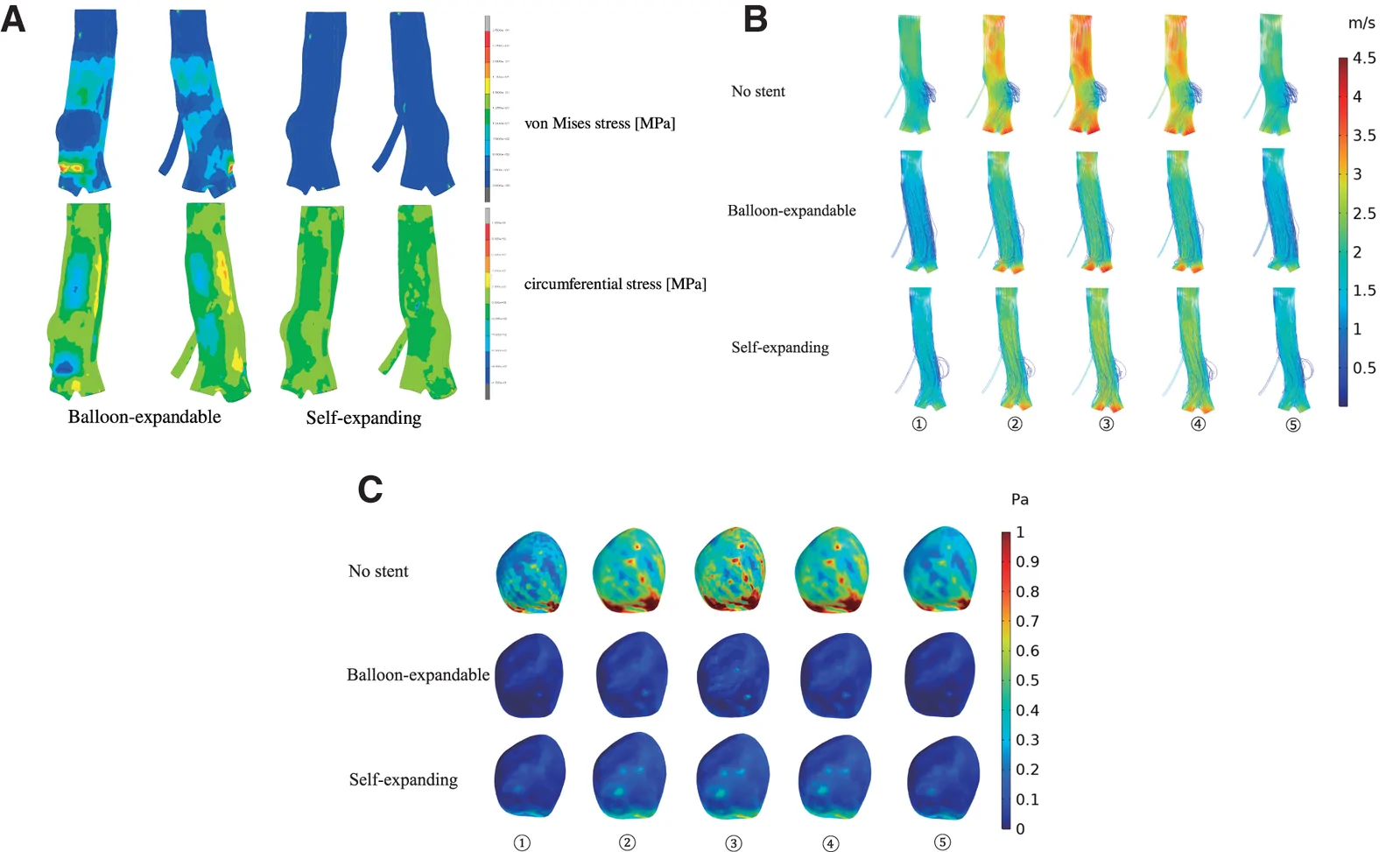
Fig. 4 Mechanical and hemodynamic changes after stent placement. Positive and negative values indicate tensile and compressive circumferential stress, respectively. (**A**) Stress distribution after stent placement. The left panels show the balloon-expandable stent model, and the right panels show the self-expanding stent model. The upper panels show von Mises stress, and the lower panels show circumferential stress. Localized stress concentrations were observed at regions of vessel curvature and diameter transition in the balloon-expandable stent model, whereas stress was distributed more uniformly in the self-expanding stent model. (**B**) Streamlines at the 5 representative phases of the cardiac cycle. The top, middle, and bottom rows show the no-stent, balloon-expandable stent, and self-expanding stent models, respectively. Flow entering the aneurysm sac decreased after placement of either stent, with straighter streamlines in the balloon-expandable stent model. (**C**) WSS distributions at the 5 representative phases. The top, middle, and bottom rows show the no-stent, balloon-expandable stent, and self-expanding stent models, respectively. WSS decreased after placement of either stent, with a more pronounced reduction around the aneurysm in the balloon-expandable stent model. WSS, wall shear stress

### FSI analysis

FSI analyses at the 5 representative phases of the cardiac cycle showed that, compared with the no-stent model, both the balloon-expandable and self-expanding stents reduced disturbed flow entering the aneurysm sac and decreased the mean intra-aneurysmal flow velocity (**Figs. 4B** and **5A**). Differences between the 2 stent types in streamline patterns and mean intra-aneurysmal flow velocity were relatively small.

**Figure 5:**
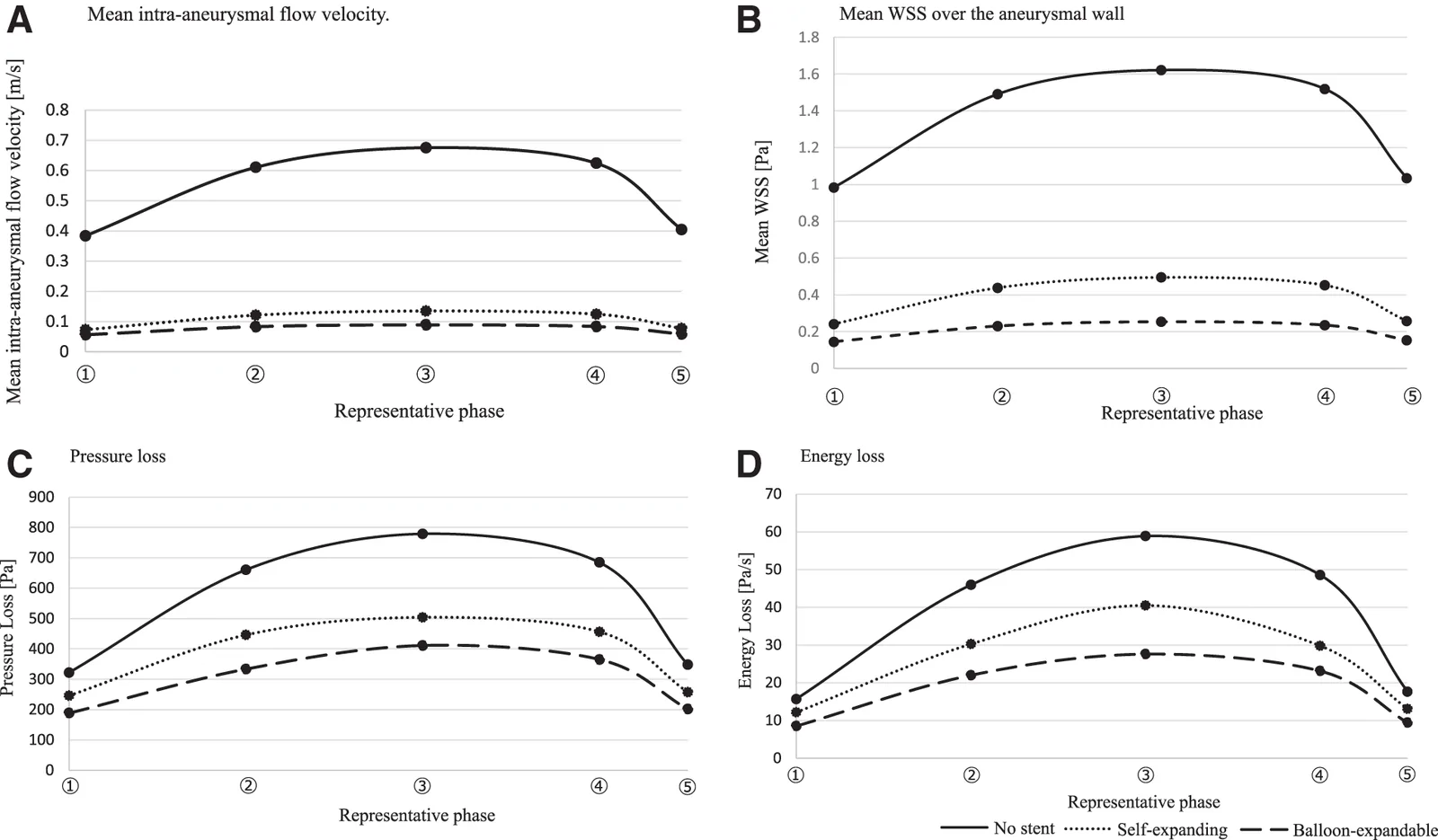
Fig. 5 Hemodynamic indices before and after stent placement. (**A**) Mean intra-aneurysmal flow velocity. (**B**) Mean WSS over the aneurysmal wall. (**C**) Pressure loss. (**D**) Energy loss. Each parameter was evaluated at the 5 representative phases defined in **Fig. 3C**. WSS, wall shear stress

WSS over the aneurysmal wall decreased following the placement of both stents. With the balloon-expandable stent, the reduction was relatively uniform over a broad area surrounding the aneurysm. In contrast, although WSS also decreased overall with the self-expanding stent, some regions showed smaller reductions than those observed with the balloon-expandable stent (**Figs. 4C** and **5B**).

Reductions in pressure loss and energy loss were also noted after the placement of both stents; however, the balloon-expandable stent exhibited greater reductions compared with the self-expanding stent (**Fig. 5C** and **5D**).

## Discussion

In this study, we compared balloon-expandable and self-expanding stents using the same saccular abdominal aortic aneurysm model under identical analysis conditions. We found that the 2 stent types had different effects on the mechanical loading of the vessel wall and on hemodynamic characteristics. Compared with the self-expanding stent, the balloon-expandable stent produced greater localized wall stress but greater reductions in WSS, pressure loss, and energy loss. The self-expanding stent produced a smaller increase in wall stress, but the changes in hemodynamic indices were also smaller than those observed with the balloon-expandable stent. These findings indicate that the ability to produce larger changes in blood flow and the ability to maintain lower mechanical loading of the vessel wall are not necessarily features of the same stent.

The differences observed between the 2 stents may be related to their structural and mechanical properties and to the resulting differences in vessel geometry after deployment.^1–3)^ In the balloon-expandable stent model, localized wall stress concentrations were observed at regions of vessel curvature and diameter transition, and the streamlines became straighter after stent placement. In contrast, the self-expanding stent conformed more closely to the vessel geometry, and wall stress was distributed more uniformly. These differences in post-deployment vessel geometry and blood flow pathways may have contributed to the differences in WSS, pressure loss, and energy loss between the 2 stent models. However, because the 2 stents differed in several characteristics, including structure, wire thickness, and mechanical properties, the contribution of each individual factor could not be determined separately in the present analysis.

Previous computational studies have shown that different stent designs can have different effects on local blood flow and WSS.^3,16)^ In the present study, we compared both hemodynamic changes and mechanical loading of the vessel wall between balloon-expandable and self-expanding stents using the same patient-specific vascular model under identical analysis conditions. The balloon-expandable stent produced greater changes in WSS, pressure loss, and energy loss, but also generated greater localized wall stress. In contrast, the self-expanding stent produced a smaller increase in wall stress but smaller changes in hemodynamic indices. Thus, a stent that produces larger changes in blood flow does not necessarily impose less mechanical load on the vessel wall. Evaluation based only on hemodynamic indices may therefore fail to fully capture the mechanical effects of a stent on the vessel wall. When assessing stent–vessel interactions, both hemodynamic effects and mechanical effects on the vessel wall should be considered. Whether this combined mechanical and hemodynamic evaluation framework is applicable to other vascular geometries and clinically used stent settings should be examined in future studies.

In this study, FSI analyses were performed separately at 5 representative phases selected from the pressure-difference waveform rather than continuously throughout the cardiac cycle. Analysis limited to representative phases may reduce the computational burden while incorporating changes in the pressure difference during the cardiac cycle. However, because the present study did not directly compare the 5-phase approach with a continuous cardiac-cycle analysis, the extent to which the 5 representative phases reproduce changes over the entire cardiac cycle remains unknown. Future studies should compare continuous and 5-phase analyses in the same model and quantitatively assess differences in flow velocity, WSS, pressure loss, energy loss, and other relevant parameters. If sufficient agreement between the 2 approaches is demonstrated, the 5-phase method may provide a less computationally demanding approach for analyzing multiple cases and different vascular geometries.

This study has several limitations. First, the analysis was based on a saccular abdominal aortic aneurysm model derived from a single patient. Therefore, it remains unclear whether the differences observed between the balloon-expandable and self-expanding stents would also be observed in other vascular geometries or vascular lesions. Further studies using multiple cases and different vascular geometries are required. Second, rather than analyzing the entire cardiac cycle continuously, we performed separate analyses at 5 representative phases. Direct comparison with continuous cardiac-cycle analysis is needed to determine how well this approach reproduces hemodynamic changes throughout the cardiac cycle. Third, this study was based solely on numerical analyses and was not validated using experimental models or in vivo studies. Fourth, the analysis model included several simplifications. The vascular geometry was constructed from CT images obtained at a single phase, and the stent and vessel wall were treated as a unified structure in the FSI analysis; therefore, changes in contact between the stent and vessel wall during the cardiac cycle were not evaluated. Finally, this study did not evaluate long-term vascular responses or clinical outcomes, such as thrombus formation, vessel wall remodeling, changes in aneurysm size, or rupture. Thus, the present study evaluated the mechanical loading of the vessel wall and hemodynamic characteristics after placement of the 2 stent types, whereas long-term vascular responses and clinical outcomes require further investigation.

## Conclusion

In this saccular abdominal aortic aneurysm model, balloon-expandable and self-expanding stents had different effects on the mechanical loading of the vessel wall and on hemodynamic characteristics. Compared with the self-expanding stent, the balloon-expandable stent produced greater reductions in WSS, pressure loss, and energy loss, but also generated greater localized wall stress. The self-expanding stent produced a smaller increase in wall stress, whereas the changes in hemodynamic indices were also smaller. These findings indicate that the ability to produce larger changes in blood flow and the ability to maintain lower mechanical loading of the vessel wall do not necessarily coexist in the same stent. Evaluation of stent–vessel interactions should therefore consider both hemodynamic effects and mechanical effects on the vessel wall.

## Supplementary Materials

Supplementary Fig. 1 Workflow of the analysis. Step 1: Construction of an aortic model from computed tomography images of a saccular abdominal aortic aneurysm. Step 2: Design of balloon-expandable stent (BES) and self-expanding stent (SES) models. Step 3: Alignment of the stent and aortic models, followed by contact analysis to evaluate stress distribution. Step 4: Construction of post-stent placement models. Step 5: Creation of the fluid domain. Step 6: FSI analyses at five representative phases selected from the pressure-difference waveform over the cardiac cycle. Step 7: Calculation of hemodynamic indices, including WSS, pressure loss, and energy loss.

Supplementary Table 1 Material properties used in the contact analysis.
