# Supplementary material for: Mechanical and Hemodynamic Effects of Balloon-Expandable and Self-Expanding Stents in a Saccular Abdominal Aortic Aneurysm Model: Fluid–Structure Interaction Analyses
Source: Ann Vasc Dis. 2026 Oct 23;19(1):26-00052. doi: 10.3400/avd.oa.26-00052 (PMC13710210; doi:10.3400/avd.oa.26-00052)
Supplement: Supplementary file 1 [file avd-19-1-26-00052-s001.pdf]

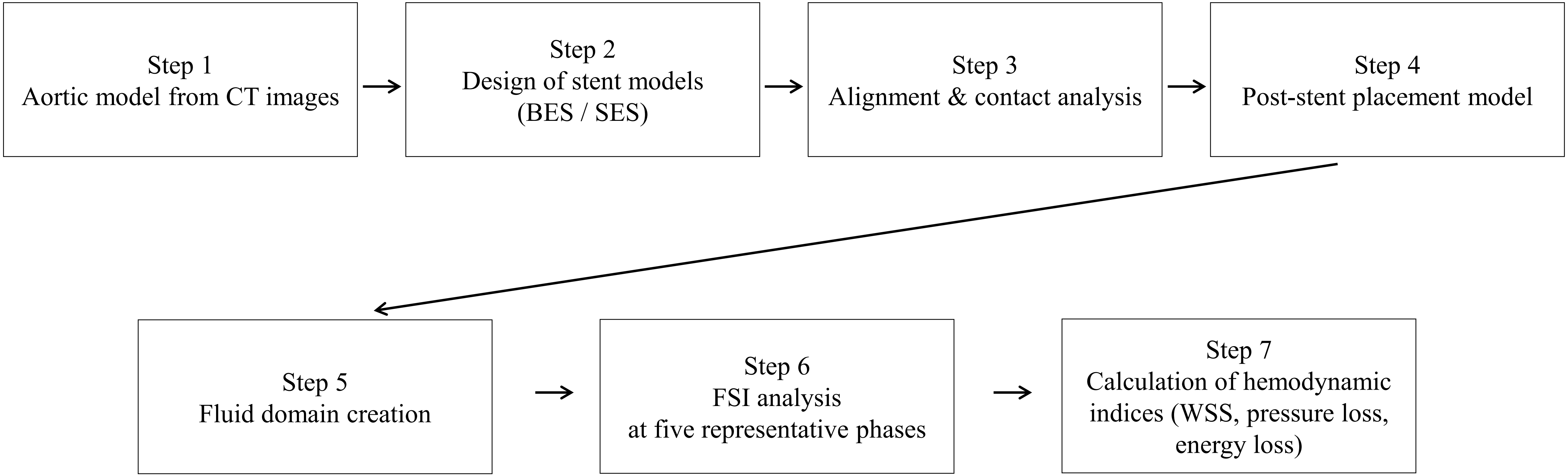
Step 1
Aortic model from CT images
Step 2
Design of stent models
(BES / SES)
Step 3
Alignment & contact analysis
Step 4
Post-stent placement model
Step 5
Fluid domain creation
Step 6
FSI analysis
at five representative phases
Step 7
Calculation of hemodynamic
indices (WSS, pressure loss,
energy loss)
