# Supplementary material for: Mechanical and Hemodynamic Effects of Balloon-Expandable and Self-Expanding Stents in a Saccular Abdominal Aortic Aneurysm Model: Fluid–Structure Interaction Analyses
Source: Ann Vasc Dis. 2026 Oct 23;19(1):26-00052. doi: 10.3400/avd.oa.26-00052 (PMC13710210; doi:10.3400/avd.oa.26-00052)
Supplement: Supplementary file 2 [file avd-19-1-26-00052-s002.pdf]

Supplementary Table 1 Material properties used in the contact analysis

Property	Aortic wall	Balloon-expandable stent	Self-expanding stent
Material	Aortic wall tissue	Nitinol	Nitinol
Young's modulus (MPa)	0.5	21000	10000
Poisson's ratio	0.49	0.33	0.33
Density (kg/m^3)	1000	6450	6450
